# Women’s characteristics and care outcomes of caseload midwifery care in the Netherlands: a retrospective cohort study

**DOI:** 10.1186/s12884-020-03204-3

**Published:** 2020-09-07

**Authors:** Pien Offerhaus, Suze Jans, Chantal Hukkelhoven, Raymond de Vries, Marianne Nieuwenhuijze

**Affiliations:** 1 Research Centre for Midwifery Science, Midwifery Education and Studies Maastricht, ZUYD University, Universiteitssingel 60, 6229 ER Maastricht, the Netherlands; 2 TNO, Department of Child Health, Schipholweg 77, 2316 ZL Leiden, The Netherlands; 3 Perined, Mercatorlaan 1200, 3528 BL Utrecht, the Netherlands; 4 CAPHRI (School for Public Health and Primary Care), Maastricht University, PO Box 616, 6200 MD Maastricht, the Netherlands; 5 Center for Bioethics and Social Sciences in Medicine, University of Michigan Medical School, 2800 Plymouth Road, Building 14, CBSSM, Ann Arbor, MI 48109-2800 USA

**Keywords:** Continuity of care, Caseload midwifery, Primary health care, Birth outcomes

## Abstract

**Background:**

The maternity care system in the Netherlands is well known for its support of community-based midwifery. However, regular midwifery practices typically do not offer caseload midwifery care – one-to-one continuity of care throughout pregnancy and birth. Because we know very little about the outcomes for women receiving caseload care in the Netherlands, we compared caseload care with regular midwife-led care, looking at maternal and perinatal outcomes, including antenatal and intrapartum referrals to secondary (i.e., obstetrician-led) care.

**Methods:**

We selected 657 women in caseload care and 1954 matched controls (women in regular midwife-led care) from all women registered in the Dutch Perinatal Registry (Perined) who gave birth in 2015. To be eligible for selection the women had to be in midwife-led antenatal care beyond 28 gestational weeks. Each woman in caseload care was matched with three women in regular midwife-led care, using parity, maternal age, background (Dutch or non-Dutch) and region. These two cohorts were compared for referral rates, mode of birth, and other maternal and perinatal outcomes.

**Results:**

In caseload midwifery care, 46.9% of women were referred to obstetrician-led care (24.2% antenatally and 22.8% in the intrapartum period). In the matched cohort, 65.7% were referred (37.4% antenatally and 28.3% in the intrapartum period). In caseload care, 84.0% experienced a spontaneous vaginal birth versus 77.0% in regular midwife-led care. These patterns were observed for both nulliparous and multiparous women. Women in caseload care had fewer inductions of labour (13.2% vs 21.0%), more homebirths (39.4% vs 16.1%) and less perineal damage (intact perineum: 41.3% vs 28.2%). The incidence of perinatal mortality and a low Apgar score was low in both groups.

**Conclusions:**

We found that when compared to regular midwife-led care, caseload midwifery care in the Netherlands is associated with a lower referral rate to obstetrician-led care – both antenatally and in the intrapartum period – and a higher spontaneous vaginal birth rate, with similar perinatal safety. The challenge is to include this model as part of the current effort to improve the quality of Dutch maternity care, making caseload care available and affordable for more women.

## Background

A solid evidence base exists for midwife-led continuity of care (MLCC) models, including caseload midwifery [1–3]. According to the Cochrane review on this topic, women in caseload midwifery or other MLCC models of care are likely to receive fewer interventions, with comparable or even better outcomes, compared to women in shared care models [3]. In this study we define caseload midwifery care as a MLCC model in which one-to-one continuity of care throughout pregnancy, childbirth and the postpartum period is guaranteed by a single midwife, with backup provided by a partner midwife and in good collaboration with other professionals. A full-time caseload midwife typically provides care to approximately 35–45 women per year [3]. The way caseload care is defined and organised varies with the context of the existing maternity care system. Caseload midwives may be self-employed or employed by a hospital or a cooperation such as one2one.org in the United Kingdom (UK). Achieving better continuity of care by implementing caseload midwifery has gained attention in various countries and maternity care systems, such as the United Kingdom [4]; Denmark [5]; Sweden [6], Australia [7]. In New Zealand, caseload midwifery is the predominant model of maternity care [8].

Caseload midwifery is not the dominant model in Dutch maternity care. Most women do not experience midwife-led continuity of care throughout the antenatal, natal and postnatal period [9]. Primary care midwives provide care to the vast majority of women, but if complications arise or are expected, women are referred to obstetrician-led care and the maternity care team in the hospital [10, 11]. Obstetric interventions such as pharmaceutical pain relief, induction or augmentation of labour are only available after a referral to obstetrician-led care. Although midwives can choose to stay involved in the care, especially after a referral during labour, these referrals usually lead to discontinuity of care, giving rise to less positive experiences [12, 13] and loss of sense of control [14]. Referral rates in the Netherlands have been rising since the eighties [10, 15, 16]. Current referral rates during labour are higher in comparison with primary midwifery care in the UK [17], without clear benefits for health outcomes [15].

Although primary maternity care in the Netherlands is midwife-led, relational continuity of care in primary midwife-led care is limited. Primary care midwives are mainly self-employed, and practice in teams of three to five midwives or more [18]. On average, a primary midwifery care practice is involved in the care of 90 to 100 women annually per participating midwife [19]. Midwives typically work in shifts of 24 or 12 h, sharing antenatal, natal, and postnatal care for the complete caseload of the practice with their team. For example, a woman who is cared for in a practice with a team of three midwives may have seen all three midwives antenatally. Each midwife in such a practice will give her share of the antenatal consultations to most of the 300 pregnant women who attend this practice annually. A woman who is referred to obstetrician-led care antenatally usually will not see her own midwife during childbirth. A woman who is still in primary midwife-led care when her labour starts, does not know which of the three midwives in the practice will attend her birth. During holidays, this could be an unknown substitute midwife. Altogether, this means that many women cared for in a group practice – which we will refer to as “regular primary midwifery care” – do not receive one-to-one continuity of care.

As a result of their preference to provide one-to-one continuity of care to a smaller number of clients, some primary care midwives in the Netherlands changed the way they organized their practices and began to offer caseload midwifery care. In our ongoing interview study with these midwives, we are learning more about their practices and motivation (Offerhaus PM, Jans S, Nieuwenhuijze MJ. The perspective of Dutch caseload midwives on their model of care. In preparation). Caseload midwives may offer extra antenatal consultations, and usually agree to be present at birth even if a referral is made and an obstetrician is the lead caregiver. The desire to offer caseload midwifery care seems to emerge from a personal desire to provide truly woman-centred care with a more personal and continuous service to pregnant women [20–23]. Caseload midwifery meets the need of women in search of a “personal” midwife, sometimes because of a concern that certain wishes will be denied in the course of regular care [24]. For instance, some women entrust their care to a caseload midwife because they prefer a homebirth despite receiving medical advice to birth in an obstetrician-led setting [25].

Apart from these observations, little is known about caseload midwifery care in the Netherlands. There is no formal description or definition of caseload midwifery care in the Dutch setting, and an accurate estimate of the actual number of caseload midwives does not exist. Furthermore, the outcomes of this type of care have not been evaluated. One study suggests that women in small midwifery practices run by one or two midwives experience fewer intrapartum referrals and fewer interventions compared to women in group practices [26]. This study by Fontein confirms that women in small practices know their midwife better, are more often accompanied by their own midwife throughout birth, even when referred to obstetrician-led care, and are more satisfied with the care received. However, this study surveyed women in midwife-led care at the start of labour and did not specifically address caseload midwifery care or the care given throughout pregnancy and birth. If caseload midwifery care is to be sustained or even expanded within the Dutch maternity care system, evaluation of this type of care is essential. The objective of our study is to contribute to this evaluation by describing the outcomes of caseload midwifery care compared to regular midwife-led care in the Netherlands in terms of maternal and perinatal outcomes, and antenatal and intrapartum referrals to obstetrician-led care.

## Methods

### Study population

The data used in our study came from the Netherlands Perinatal Registry (Perined). The registry routinely collects and combines data on antenatal, intrapartum and postnatal care from four separate national registries; one for primary midwife-led care (LVR1), one for maternity care by general practitioners (LVR1h), one for obstetrician-led care (LVR2), and one for neonatal care (LNR). The Netherlands Perinatal Registry contains data on approximately 98% of all births in the Netherlands [16]. We selected all women who gave birth in 2015 and received antenatal care in a primary midwifery practice in the Netherlands. Exclusion criteria were birth at a gestational age under 28 weeks, or an antenatal transfer to secondary or tertiary care for pregnancy complications before 28 weeks. In this way we ensured that women experiencing serious medical problems in early pregnancy are not included. The study population, being in primary midwife-led care at 28 weeks of pregnancy, is, by definition, considered to be low risk.

### Identification of caseload women

Caseload midwives throughout the Netherlands were identified with a snowball method, starting from the professional network of the first author (PO). All potential participants were contacted and were included if their practice description fit the caseload definition [3], based on a short questionnaire. Midwives working in a team or as a couple were included only if they offered 24/7 one-to-one continuity of care throughout pregnancy and birth. Midwives working according to an agreed schedule or duty roster and who shared their clients with their colleagues – a customary arrangement in regular midwifery practices - were excluded from the caseload group. Participating caseload midwives gave informed consent and voluntary disclosed their LVR1 practice registration number, to enable the researchers to anonymously identify caseload clients in the study population.

### Identification of matched controls

Based on personal communication with caseload midwives, we expected their clients to be more often multiparous, older, with a higher social-economic status (SES) and more often of a Dutch background, compared to women in regular care. To reduce confounding and enhance comparability of characteristics in the study population, we matched each woman in caseload care to three women in regular midwife-led care. We used parity (0, 1 or more), maternal age (six categories) and background (Dutch or non-Dutch) as matching variables. Since SES is measured on neighborhood level only in the Perined database, we decided not to use SES as a matching variable. Because serious regional variation in maternity care has been described in the Netherlands [27, 28] we also included the postal code (first two out of four digits) in the matching procedure to minimize confounding based on these regional differences. Exact matching was performed randomly using SPSS for Windows, version 22.

### Characteristics and outcome variables

The following maternal characteristics were collected from the database: maternal age; parity; ethnic background (Dutch or non-Dutch); SES and level of urbanisation of the neighbourhood in which women were living were determined using the four digits of the postal code [29]. The characteristics of birth we collected were: planned and actual place of birth, gestational age at birth, birthweight, multiple pregnancy and foetal presentation at birth. To explore whether women in caseload care may have switched to a caseload midwife at a later stage after starting their care in another midwifery practice or a secondary care hospital team [25], we also collected the gestational age at intake in the practice that recorded the birth.

The primary outcomes of interest were antenatal or intrapartum referral to obstetrician-led care and mode of birth (spontaneous vaginal, instrumental vaginal or caesarean). Other outcomes of interest were inductions of labour, maternal morbidity (blood loss and perineal trauma) and perinatal outcomes (perinatal mortality and Apgar score). In a sub-analysis among low risk women who were in midwife-led care at the start of labour, we also observed interventions during childbirth (augmentation of labour, pharmaceutical pain relief and episiotomy).

### Analysis

All analyses of outcomes are presented separately for nulliparous and multiparous women. In the main analysis, we used inferential statistics (Chi square; two-sided t-test) to compare the outcomes for women in caseload care and regular care. To capture the differences between intrapartum care in caseload and regular practices, we performed an exploratory sub-analysis among low risk women who started labour in midwife-led care. For this sub-analysis we excluded all women who experienced an antenatal referral after 28 weeks and women with the following risk factors: gestational age < 37 or > 42 weeks, non-vertex presentation, and multiple pregnancy. Given these exclusions, we could no longer use our matching procedure; for this reason we used only descriptive statistics (percentages) to illustrate the differences between the two groups.

## Results

We identified 33 midwives that fulfilled the description of caseload midwifery care. Among them 23 had a LVR1 practice registration number in 2015, individually or together with other caseload midwives. Six midwives started practice registration in 2016, and another four did not provide a practice registration number. These 10 were excluded from the study.

### Characteristics of caseload clients

The national database contained the records of 127,818 women in midwife-led care at a gestational age of 28 weeks and beyond. Of these women, we included all 657 women that were registered by caseload midwives in our study. The matched cohort contained 1954 women in regular midwifery care. If the matching procedure had been fully successful, the matched cohort would have contained 1971 controls. This means we missed 17 controls (0,9%). All cases had at least one exact match.

Women in caseload care were slightly older (mean 31.6 vs 30.4 year) when compared to women in the total national cohort, they more often had a Dutch background (79.5% vs 75.6%), and were somewhat more often nulliparous (48.6% vs 45.8%). These and other characteristics are shown in Table 1.

**Table 1:** Maternal and pregnancy characteristics of women in primary midwifery care at 28 weeks and beyond

		National		Caseload practices		Matched controls		*p-value (caseload* vs *matched)*	
		*n* = 127,818		*n* = 657		*n* = 1954			
**Maternal age**								*na*	^a^
	Mean	30.4	(SD 4.6)	31.6	(SD 4.5)	31.4	(SD 4.4)		
	< 25	13,197	(10.3%)	32	(4.9%)	99	(5.1%)		
	25–29	41,138	(32.2%)	192	(29.2%)	567	(29.0%)		
	30–34	49,438	(38.7%)	272	(41.4%)	814	(41.7%)		
	35–39	20,885	(16.3%)	132	(20.1%)	393	(20.1%)		
	≥40	3160	(2.5%)	29	(4.4%)	81	(4.1%)		
**Parity**								*na*	^a^
*(missing: 2)*	0	58,608	(45.8%)	319	(48.6%)	940	(48.1%)		
	1	46,010	(36.0%)	240	(36.5%)	722	(36.9%)		
	≥2	23,198	(18.1%)	98	(14.9%)	292	(14.9%)		
**Background**								*na*	^a^
	Dutch	96,589	(75.6%)	522	(79.5%)	1555	(79.6%)		
	Non Dutch	31,229	(24.4%)	135	(20.5%)	399	(20.4%)		
**SES**								*0.019*	^b^
*(missing 574)*	1 (low)	41,146	(32.3%)	237	(36.7%)	757	(39.0%)		
	2 (intermediate)	55,949	(44.0%)	248	(38.4%)	802	(41.4%)		
	3 (high)	30,149	(23.7%)	160	(24.8%)	380	(19.6%)		
**Urbanisation**								*< 0.001*	^b^
*(missing 206)*	Very urban	31,693	(24.8%)	184	(28.1%)	539	(27.6%)		
	Urban	32,707	(25.6%)	193	(29.5%)	360	(18.4%)		
	Semi-urban	23,989	(18.8%)	65	(9.9%)	253	(13.0%)		
	Rural	21,991	(17.2%)	93	(14.2%)	467	(23.9%)		
	Very Rural	17,232	(13.5%)	119	(18.2%)	333	(17.1%)		
**Gestational age at intake in practice**								*< 0.001*	^b^
*(missing 1206)*	≤12	112,767	(89.1%)	480	(73.2%)	1770	(90.6%)		
	13–27	10,578	(8.4%)	101	(15.4%)	131	(6.7%)		
	28–36	2763	(2.2%)	57	(8.7%)	37	(1.9%)		
	≥37 wk	506	(0.4%)	18	(2.7%)	3	(0.2%)		
**Antenatally planned place of birth**								*< 0.001*	^b^
*(missing 3269)*	home	27,111	(21.8%)	291	(44.5%)	387	(20.3%)		
	birthcentre	12,666	(10.2%)	28	(4.3%)	87	(4.6%)		
	hospital	70,322	(56.5%)	272	(41.6%)	1177	(61.6%)		
	undecided	14,450	(11.6%)	63	(9.6%)	259	(13.6%)		
**Gestational age at birth in weeks**								*< 0.001*	^b^
*(missing 1432)*	< 37^0^	5121	(4.1%)	20	(3.1%)	80	(4.1%)		
	37–41^6^	119,429	(94.5%)	601	(91.8%)	1829	(94.2%)		
	≥42^0^	1836	(1.5%)	34	(5.2%)	32	(1.6%)		
	Mean	39.3	(SD 1.5)	39.5	(SD 1.6)	39.3	(SD 1.5)	*0.004*	^c^
**Birthweight (gram)**									
*(missing 796)*	Mean	3469	(SD 513)	3.495	(SD 499)	3.475	(SD 508)	*0.382*	^c^
	SGA (<p5)	4575	(3.6%)	26	(4.0%)	61	(3.1%)	*0.302*	^b^
	LGA (>p95)	5765	(4.5%)	26	(4.0%)	82	(4.2%)	*0.790*	^b^
**Birthweight Categories**								*0.424*	^b^
	< 2500	4267	(3.3%)	12	(1.8%)	52	(2.7%)		
	2500–4500	120,844	(94.7%)	630	(96.0%)	1863	(95.3%)		
	> 4500	2459	(1.9%)	14	(2.1%)	35	(1.8%)		
**Multiple pregnancy**		135	(0.1%)	1	(0.2%)	3	(0.2%)		
**Non-vertex presentation at birth**	breech	3881	(3.0%)	15	(2.3%)	58	(3.0%)		
	other / unknown	2746	(2.1%)	8	(1.2%)	42	(2.1%)		

^a^*not applicable, matching variable*

^b^*Chi Square*

^c^*Two-sided t-test*

After matching for parity, age and background, women in caseload care showed some differences in other demographic characteristics with the women in regular care. They more often lived in a neighbourhood with a higher SES (24.8% vs 19.6%), more often in an urban neighbourhood (29.5% vs 18.4%) and less often in a rural neighbourhood (14.2% vs 23.9%). There were no significant differences in mean birthweight, birthweight categories, small for gestational age (<percentile 5) or large for gestational age (>percentile 95).

Other characteristics seem to confirm that women in caseload care are a distinct group. They more often opted for a home birth (44.5%% vs 20.3%), and more often had a late intake in the practice (11.4% vs 2.1% at ≥28 weeks), likely an indication of a switch in care provider during antenatal care. Gestational age at birth was slightly higher (39.5 vs 39.3 weeks; *p*-value 0.004), and more women in caseload care gave birth at or after 42 + 0 weeks (5.2% vs 1.6%). This suggests that they more often opted for expectant management in case of a prolonged pregnancy, despite existing recommendations in the Netherlands [29].

### Primary outcomes

Outcomes of women in caseload care were compared to the matched cohort (Table 2). We found that women in caseload care were less often referred to obstetrician-led care (p-value < 0.001). A small majority (53.1%) stayed in primary midwife-led care without a referral to obstetrician-led care (nulliparous women: 40.3%; multiparous women: 65.2%), compared to 34.3% in the matched cohort (nulliparous women 23.8%; multiparous women: 44.2%). The overall referral rate was 46.9% in the caseload group and 65.7% in the matched cohort. A lower referral rate was observed both in the antenatal period and in the intrapartum period, for nulliparous and for multiparous women. In the antenatal period, 24.2% of women in caseload care experienced a referral to obstetrician-led care and 22.8% were referred intrapartum, versus 37.4 and 28.3% in regular care.

**Table 2:** Outcomes for women in caseload midwifery care and regular midwifery care

	All women				*p-value Chi square*	Nulliparous women				Multiparous women			
	Caseload practices		Matched cohort			Caseload practices		Matched cohort		Caseload practices		Matched cohort	
	*n* = 657		*n* = 1954			*n* = 319		*n* = 940		*n* = 338		*n* = 1014	
**Referrals**					*< 0.001*								
no referral	347	53.1%	666	34.3%		128	40.3%	222	23.8%	219	65.2%	444	44.2%
antenatal	158	24.2%	725	37.4%		77	24.2%	328	35.1%	81	24.1%	397	39.5%
intrapartum	149	22.8%	548	28.3%		113	35.5%	384	41.1%	36	10.7%	164	16.3%
*(missing: 18)*													
**Mode of birth**					*0.001*								
spontaneous vaginal	552	84.0%	1492	77.0%		240	75.2%	636	68.3%	312	92.3%	856	85.0%
vaginal instrumental	43	6.5%	170	8.8%		36	11.3%	146	15.7%	7	2.1%	24	2.4%
caesarean section	62	9.4%	276	14.2%		43	13.5%	149	16.0%	19	5.6%	127	12.6%
*(missing: 16)*													
**Start of labour**					*< 0.001*								
Spontaneous	550	83.7%	1431	73.2%		263	82.4%	709	75.4%	287	84.90%	722	71.2%
Induction	87	13.2%	410	21.0%		47	14.8%	187	19.9%	40	11.8%	223	22.0%
*amniotomy only*	*19*	*2.9%*	*78*	*4.0%*		*6*	*1.9%*	*27*	*2.9%*	*13*	*3.80%*	*51*	*5.0%*
*hormonal/foley/oxytocin*	*68*	*10.4%*	*332*	*17.0%*		*41*	*12.9%*	*160*	*17.0%*	*27*	*8.00%*	*172*	*17.0%*
Caesarean section	20	3.0%	113	5.8%		9	2.8%	44	4.7%	11	3.30%	69	6.8%
**Actual place of birth**					*< 0.001*								
home	259	39.4%	314	16.1%		92	28.8%	95	10.1%	167	49.4%	219	21.6%
birthcentre	14	2.1%	31	1.6%		6	1.9%	12	1.3%	8	2.4%	19	1.9%
hospital (primary care)	72	11.0%	318	16.3%		28	8.8%	113	12.0%	44	13.0%	205	20.2%
hospital (secondary care)	312	47.5%	1291	66.1%		193	60.5%	720	76.6%	119	35.2%	571	56.3%
**Maternal outcomes**													
PPH > 1000 ml	33	5.0%	135	6.9%	*0.088*	22	6.9%	78	8.3%	11	3.3%	57	5.6%
intact perineum *(vaginal births)*	246	41.3%	468	28.2%	*< 0.001*	85	30.8%	164	21.0%	161	50.5%	304	34.5%
Episiotomy *(vaginal births)*	95	16.0%	438	26.4%	*< 0.001*	74	26.8%	336	43.0%	21	6.6%	102	11.6%
3rd/4th perineal tear *(vaginal births)*	14	2.4%	47	2.8%	*0.687*	12	4.3%	35	4.5%	2	0.6%	12	1.4%
**Perinatal outcomes**													
Perinatal mortality	1	0.2%	7	0.4%	*0.408*	1	0.3%	5	0.5%	0	–	2	0.2%
Apgar 5 min < 7	8	1.2%	35	1.8%	*0.318*	6	1.9%	21	2.2%	2	0.6%	14	1.4%

Mode of birth also differed between women in caseload care and in the matched cohort (*p*-value 0.001). Women in caseload care more often (84.0% vs 77.0%) experienced a spontaneous vaginal birth (nulliparous women: 75.2% vs 68.3%, multiparous women: 92.3% vs 85.0%), and less often (9.4% vs 14.2%) a caesarean section (nulliparous women: 13.5% vs 16.0%, multiparous women: 5.6% vs 12.6%).

### Other outcomes

Interventions at the start of labour differed between the two groups (*p*-value < 0.001). Women in caseload care were more likely to experience a spontaneous start of labour (83.7%) compared to women in regular care (73.2%). They were less often induced (13.2% vs 21.0%) and less often had an elective caesarean section (3.0% vs 5.8%). A similar pattern existed for nulliparous and multiparous women. The place of birth was also different (p-value 0.001). A larger proportion of women in caseload care had an out-of-hospital birth (39.4% at home and 2.1% in a birth centre) compared to women in regular care (16.1% at home and 1.6% in a birth centre). More multiparous women than nulliparous women had an out-of-hospital birth, both in caseload practices and in regular care. The larger proportion of hospital births in obstetrician-led care in regular care (66.1% vs 47.5%) reflects the higher referral rate discussed earlier. A postpartum haemorrhage (> 1000 ml) occurred in 5.0% (nulliparous women 6.9%; multiparous women 3.3%) versus 6.9% in the regular care group (nulliparous women 8.3%; multiparous women 5.6%). This difference was not statistically significant (*p*-value 0.088). Fewer women in caseload care experienced an episiotomy (16.0% vs 26.4%, p-value < 0.001) and more had an intact perineum (41.3% vs 28.2%; p-value < 0.001). There was no significant difference in 3rd or 4th degree perineal ruptures (2.4% vs 2.8%; p-value 0.687). Nulliparous women were more likely to experience perineal damage compared to multiparous women, both in the caseload group and in the matched cohort.

Unfavourable perinatal outcomes were rare in both groups. Perinatal mortality occurred in one case (0.2%) in the caseload group and in seven cases (0.4%) in the matched cohort. A low Apgar score (< 7 at 5 min) was registered in 8 cases (1.2%) in the caseload group and in 35 cases (1.8%) in the matched cohort. These differences were not statistically significant.

### Sub-analysis: intrapartum care

**Fig. 1:**
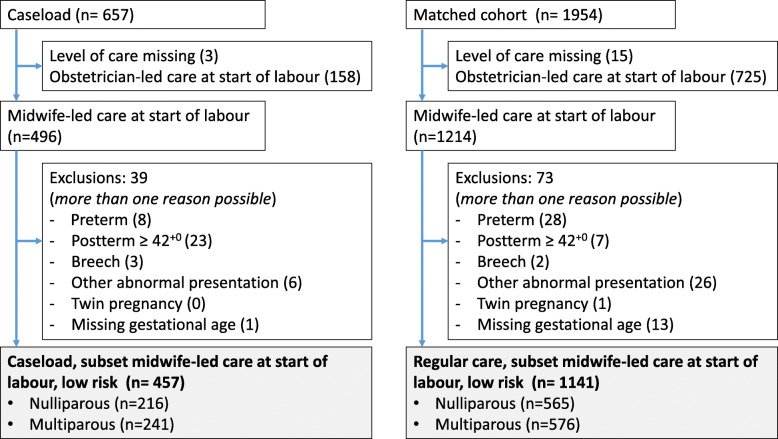
Flowchart exclusions for sub-analysis ‘midwife-led care at start of labour’

In a sub-analysis we analysed births of women who had no antenatal referral to obstetrician-led care, describing births attended by a primary care midwife in caseload care (*n* = 496) and births from the regular care group (*n* = 1214). We excluded 39 (7.9%) births with a known risk factor (23 pregnancies ≥42 weeks) from the caseload group and 73 (6.0%; 7 pregnancies ≥42 weeks) from the regular care group (see Fig. 1 for a flowchart). As a result of this selection of women without known risk factors at the start of term labour, the groups are no longer matched and the results should be interpreted with caution.

Results are displayed in Table 3. In this sub-analysis the lower intrapartum referral rate in caseload care among nulliparous women (42.6% vs 63.0%) and multiparous women (13.3% vs 25.7%) is largely explained by a lower referral rate in the first stage of labour: 24.1% vs 44.4% for nulliparous women and 11.2% vs 19.8% for multiparous women. Both nulliparous and multiparous women in caseload care experienced fewer interventions during labour, and more often had a spontaneous vaginal birth. As in the main analysis, maternal morbidity was lower in the caseload group.

**Table 3:** Outcomes in caseload and regular care for women who started labour in primary care *(37–42 wk, singleton, vertex)*

	Nulliparous women				Multiparous women			
	Caseload practices		Regular midwife-led care		Caseload practices		Regular midwife-led care	
	*n* = 216		*n* = 565		*n* = 241		*n* = 576	
**No intrapartum referral**	124	57.4%	209	37.0%	209	86.7%	428	74.3%
**Intrapartum referral**	92	42.6%	356	63.0%	32	13.3%	148	25.7%
*1st stage*	*52*	*24.1%*	*251*	*44.4%*	*27*	*11.2%*	*114*	*19.8%*
*2nd stage*	*21*	*9.7%*	*63*	*11.2%*	*3*	*1.2%*	*7*	*1.2%*
*other/ unclear*	*19*	*8.8%*	*42*	*7.4%*	*2*	*0.8%*	*27*	*4.7%*
**Interventions during labour**								
amniotomy	61	28.2%	236	42.7%	61	25.4%	289	50.8%
augmentation	51	23.8%	215	38.4%	12	5.0%	60	10.5%
pharmaceutical pain relief	42	20.8%	193	35.4%	9	3.8%	48	8.5%
episiotomy	40	18.5%	220	38.9%	8	3.3%	48	8.3%
**Mode of birth**								
spontaneous	181	83.8%	425	75.9%	234	97.1%	555	96.7%
vaginal instrumental	21	9.7%	93	16.6%	3	1.2%	11	1.9%
caesarean section	14	6.5%	42	7.5%	4	1.4%	8	1.4%
**Maternal outcomes**								
PPH > 1000 cc	15	6.9%	40	7.1%	8	3.3%	27	4.7%
Perineum intact (vaginal births only)	67	33.2%	109	21.0%	122	51.5%	201	35.5%
3rd/4th perineal tear (vaginal births only)	9	4.5%	24	4.6%	1	0.4%	7	1.2%

## Discussion

Our study shows that in the Netherlands, when compared with regular midwife-led care, caseload midwife-led care was associated with considerably fewer referrals to obstetrician-led care – both antenatally and in the intrapartum period – and with more spontaneous vaginal births. These results were found for both nulliparous and multiparous women. Furthermore, we observed fewer interventions during labour and birth and less maternal morbidity in caseload midwifery care. The incidence of perinatal mortality or a low Apgar score were low in both groups.

### Methodological considerations

The main challenge in this comparison of results from caseload care and regular midwife-led care is the comparability of women in both groups. Women were included when in primary care at 28 weeks gestation and are therefore likely to have a relatively low risk profile. Several factors may still cause differences in risk profiles, although our matching procedure successfully minimized confounding by parity, age, or background. To control for regional variation in interventions we added the first two digits of the postal code in the matching procedure. Because our measure for SES and urbanization both are based on the postal code as well, we chose not to match on these variables. Based on the somewhat higher SES in the caseload group compared to the matched controls in regular care, a slightly more favorable risk profile cannot be ruled out. We could also not describe, nor control for, risk factors such as obesity, smoking or other life style factors since these are not reliably registered in the Perined database. On the other hand, we observed no important differences in preterm births, mean birthweight, and SGA (<p5) between the national cohort, the caseload group and the matched cohort. Therefore, we assume that the risk profiles of the caseload group and the matched cohort are not very different and do not lead to important bias.

Nevertheless, women in caseload care may be different from women in regular care in other important ways that cannot easily be assessed. The observed characteristics confirm that women in caseload care are a distinct group, with a higher than average motivation for a physiological birth, including homebirth and expectant management in prolonged pregnancy beyond 42 gestational weeks. Seeking the care of a caseload midwife may be a part of this inclination [25]. It is not possible to assess to what extent these preferences resulted in continuation of primary care or opting for homebirths in situations that usually lead to referral to secondary care in the hospital. We did find a slightly higher percentage of women with known risk factors- mainly gestational age ≥ 42 weeks - in the caseload group. Altogether, the lower referral and intervention rate we found will at least partly be a result of specific preferences among this group of women.

The study is too small for a reliable comparison in perinatal mortality or serious perinatal morbidity, since these outcomes are rare. At the same time, our results do not suggest that perinatal safety is compromised in caseload care: percentages of both perinatal mortality and low Apgar scores in the caseload and control groups in the caseload and control groups were similar. In a larger, preferably prospective, study it would be interesting to analyze perinatal results with the possibility to control for several risk factors including maternal lifestyle and to audit cases with severe perinatal morbidity or mortality.

While our study has certain methodological limitations – e.g. self-selection – our results are supported by high quality studies of caseload midwifery care. For instance, randomized trials [1, 2, 30, 31] showed no differences in neonatal outcomes such as low Apgar scores or admission to neonatal intensive care. A meta-analysis showed a higher spontaneous vaginal birth rate in caseload midwifery care [3]. Observational studies of caseload midwifery in various settings consistently show fewer interventions during childbirth, without compromising perinatal safety [32–35].

### Continuity of care: empowering women for childbirth

We were not able to assess the level of one-to-one continuity of care in the caseload group because this information is not available in the Perined database. However, we are confident that the majority of the caseload clients received a high level of continuity of care throughout pregnancy and childbirth. In the inclusion procedure, participating midwives confirmed that they remained involved in the care when antenatal obstetric consultation or referral was needed, and all offered continuity of care during labour, even when there was an intrapartum referral. Most of them also inform women of this service on their practice website. Fontein [26] observed a similar pattern in practices with one or two midwives, where continuity of care was higher, and the midwife-woman relationship was experienced more positively when compared to group practices.

A recent meta-synthesis of 13 qualitative articles examining women’s perspectives on continuity of care models confirms the importance of such relationships for women, concluding that ‘the midwife–woman relationship is the vehicle through which trust is built, personalised care is provided, and the woman feels empowered’ [36]. Fontein et al. describe this type of relationship as a key feature of woman-centered care [37]. Such relationships are more likely to evolve in continuity models than in fragmented care offered in busy institutions or by large midwifery teams [38]. Indeed, caseload midwives in the Netherlands report that they offer this kind of woman-centered care [20–23] in order to strengthen women and give them more control in the care process [39]. Our data suggest that a strong woman-centered model of care – with the midwife in a supporting and facilitating role – results in more control by women themselves and a more optimal, less medicalized pregnancy and birth process, with no compromise in perinatal safety. Improving woman-centeredness is an important point in the quality improvement agenda for maternity care in the Netherlands [40]. Based on our results, finding ways to implement caseload midwifery on a larger scale should be considered.

## Conclusion

Our study found that caseload midwifery care in the Netherlands is associated with a lower referral rate to obstetrician-led care – both antenatally and in the intrapartum period – and more spontaneous vaginal births compared to regular midwife-led care, without any indications that perinatal safety is compromised. Our findings are in line with the growing body of evidence on the importance of continuity of midwife-led care [3, 41]. A larger scale prospective study is needed to provide more definitive evidence on perinatal and maternal outcomes of caseload care and to provide insights in the lower referral rate to obstetrician-led care and in the cost-effectiveness of the caseload model in the context of Dutch maternity care. Such a study should also examine women’s perspectives on this model of care in the Netherlands. If the results of a larger study mirror our findings, the challenge will be to make this model available and affordable for more women in the Netherlands.

## Data Availability

The data that support the findings of this study are available from Perined but restrictions apply to the availability of these data, which were used under license for the current study, and so are not publicly available. Data are however available from the first author upon reasonable request and with permission of Perined.

## Abbreviations

LNR: Landelijke Neonatale Registratie (National Neonatal Registry)
LVR: Landelijke Verloskundige Registratie (National Perinatal Registry)
MLCC: Midwife-Led Continuity of Care
SES: Social-economic status
UK: United Kingdom
